# Ubiquitinome Analysis Uncovers Alterations in Synaptic Proteins and Glucose Metabolism Enzymes in the Hippocampi of Adolescent Mice Following Cold Exposure

**DOI:** 10.3390/cells13070570

**Published:** 2024-03-25

**Authors:** Xin-Yue Li, Xin Yin, Jing-Jing Lu, Qian-Ru Li, Wan-Qun Xing, Qi Han, Hong Ji, Shi-Ze Li, Huan-Min Yang, Jing-Ru Guo, Zhi-Quan Wang, Bin Xu

**Affiliations:** 1 College of Animal Science and Veterinary Medicine, Heilongjiang Bayi Agricultural University, Daqing 163319, China; 15042363578@163.com (X.-Y.L.);; 2 Department of Agricultural, Food and Nutritional Science, University of Alberta, Edmonton, AB T6G 2P5, Canada

**Keywords:** cold exposure, hippocampus, ubiquitinome, PSD-95, glucose metabolism

## Abstract

Cold exposure exerts negative effects on hippocampal nerve development in adolescent mice, but the underlying mechanisms are not fully understood. Given that ubiquitination is essential for neurodevelopmental processes, we attempted to investigate the effects of cold exposure on the hippocampus from the perspective of ubiquitination. By conducting a ubiquitinome analysis, we found that cold exposure caused changes in the ubiquitination levels of a variety of synaptic-associated proteins. We validated changes in postsynaptic density-95 (PSD-95) ubiquitination levels by immunoprecipitation, revealing reductions in both the K48 and K63 polyubiquitination levels of PSD-95. Golgi staining further demonstrated that cold exposure decreased the dendritic-spine density in the CA1 and CA3 regions of the hippocampus. Additionally, bioinformatics analysis revealed that differentially ubiquitinated proteins were enriched in the glycolytic, hypoxia-inducible factor-1 (HIF-1), and 5‘-monophosphate (AMP)-activated protein kinase (AMPK) pathways. Protein expression analysis confirmed that cold exposure activated the mammalian target of rapamycin (mTOR)/HIF-1α pathway. We also observed suppression of pyruvate kinase M2 (PKM2) protein levels and the pyruvate kinase (PK) activity induced by cold exposure. Regarding oxidative phosphorylation, a dramatic decrease in mitochondrial respiratory-complex I activity was observed, along with reduced gene expression of the key subunits NADH: ubiquinone oxidoreductase core subunit V1 (*Ndufv1*) and *Ndufv2*. In summary, cold exposure negatively affects hippocampal neurodevelopment and causes abnormalities in energy homeostasis within the hippocampus.

## 1. Introduction

Cold exposure is a type of stressor that people often encounter in their daily lives. For soldiers engaged in military activities in cold environments, as well as some workers in high-altitude environments, cold exposure is a problem that affects work performance and even threatens life safety. Studies have proven that cold exposure has a certain negative impact on cognitive performance in adults, as observed in the Stroop test, Digit Span task, Sternberg Memory Search test, and so on [1,2,3,4]. Evidence also suggests that cold exposure induces anxiety behaviors in rodents [5]. The hippocampus is one of the brain regions that is most sensitive to various stressors. Chronic stress may damage the hippocampus, leading to impaired spatial memory and dysregulation of the hypothalamic–pituitary–adrenal axis (HPA) [6,7,8,9,10]. Dendritic atrophy and dendritic-spine reduction are typical stress-induced hippocampal structural changes, which are closely related to stress-induced hippocampal dysfunction [11]. Notably, as a sensitive period for brain development, adolescence is particularly vulnerable to various stressors [12]. Our previous studies focused on adolescent mice showed that cold exposure has a negative effect on hippocampal neurodevelopment in adolescent mice, leading to hippocampal neurotransmitter disorders, neuronal loss, and behavioral changes [13,14,15,16]. However, the mechanistic basis of these observations is not completely clear.

Remarkably, a link between stress-induced neuronal damage and changes in energy supply has been established [17]. The stress response has been shown to suppress brain glucose uptake [6], interfere with mitochondrial DNA expression [18,19], cause neuronal mitochondrial autophagy disorders, and affect the transfer of energy from astrocyte energy reservoirs to neurons [20], thus resulting in shortened dendritic length and decreased dendritic-spine density [21]. As we know, cold exposure presents a severe challenge to whole-body energy metabolism, as the brain is responsible for regulating energy distribution to other organ systems. The question remains: is energy metabolism of the brain affected by cold stress? Adolescence is a special period in brain development; evidence suggests that brain energy expenditure is higher in childhood than in adulthood due to the high energy expenditure required for neurodevelopmental processes [22]. The effect of cold exposure on energy metabolism in the hippocampi of adolescent mice is a matter of concern.

As a post-translational modification essential to normal cellular activities, ubiquitination has been shown to direct substrates to proteasomes and autophagosomes, as well as regulate degradation-independent processes [23]. Ubiquitin monomers can form different polyubiquitin chains through their seven internal lysine residues, which endow ubiquitination with different functions [24]. For instance, the K48-linked ubiquitin chain targets substrates for proteasome degradation, whereas K63-linked ubiquitin chains serve as signals for autophagy, endocytosis, signal transduction, and DNA repair [25,26]. During neurodevelopment, ubiquitination-dependent pathways dynamically regulate synapse formation and synapse elimination, thus controlling various processes of neural circuit assembly [27]. In response to changes in neuronal activity, crucial postsynaptic scaffold proteins such as spine-associated Rap GTPase-activating protein (SPAR), guanylate kinase-associated protein (GKAP), and Shank are directly regulated by the ubiquitin–proteasome system at the postsynaptic density (PSD) [28,29,30]. Among them, PSD-95, the most abundant scaffold protein in the PSD [31], has been proven to drive synapse maturation through a K63-polyubiquitination-dependent machinery [32]. Consistent with a central role for ubiquitin-dependent machinery in normal nervous system function, the application of proteasome inhibitors or the knockdown of key ubiquitin ligases such as RNF20 results in impaired long-term memory [33,34]. Additionally, protein ubiquitination participates in regulating energy metabolism through the modulation of mitochondrial autophagy and the energy sensor adenosine 5‘-monophosphate (AMP)-activated protein kinase (AMPK) [35,36]. However, a systematic study of the effects of chronic cold exposure on the ubiquitination of hippocampal proteins in adolescent mice is currently lacking.

In this study, we applied label-free quantitative mass spectrometry techniques to explore ubiquitinome changes in the hippocampi of adolescent mice after cold exposure. Subsequently, we demonstrated that the differentially ubiquitinated proteins were relevant to pathways that included synaptic signaling, hypoxia-inducible factor-1 (HIF-1) signaling, AMPK signaling, and glycolysis. Ubiquitinome datasets, combined with immunoprecipitation results, confirmed that PSD-95 ubiquitination is involved in the hippocampal response to chronic cold exposure. We also found that cold exposure caused abnormalities in hippocampal glucose homeostasis, inducing extensive changes in glycolysis, the TCA cycle, and oxidative phosphorylation processes within the hippocampus. The results of our ubiquitinomic analysis highlight the importance of ubiquitination modification for the hippocampal response to cold exposure, laying the groundwork for further mechanistic studies.

## 2. Materials and Methods

### 2.1. Animals and Experimental Design

Adolescent male C57BL/6 mice were provided by the Experimental Animal Center of Changsheng Biotechnology (Changchun, China). Both pre-feeding and cold-exposure experiments were carried out in the climate-controlled chamber of Heilongjiang Bayi Agricultural University. Before the experiment started, mice were pre-fed for one week. The chamber maintained an ambient temperature of 24 ± 2 °C and 40% relative humidity. The mice were subjected to a 12/12 h light/dark cycle, with lights on from 8:00 a.m. to 8:00 p.m. The mice had free access to food and water [37]. Following the pre-feeding period, CE groups (five weeks old) were transferred to a climatic chamber set at 4 °C for 3 h per day between 8:00 a.m. and 8:00 p.m. [37]. The CE mice were kept at room temperature for the rest of the day (except for 3 h of cold exposure). The chronic cold-exposure process lasted for 21 days. RT groups (five weeks old) were housed in a climate chamber maintained at 24 ± 2 °C throughout the entire experimental period. All experimental procedures were approved by the Science and Technology Ethics Committee of Heilongjiang Bayi Agricultural University (approval code: DWKJXY2022063, approved on 1 June 2022) [38].

### 2.2. Sample Collection

For immunohistochemical experiments, all mice were injected with 1.5% sodium pentobarbital. Then, they were subjected to transcardial perfusion with normal saline (NS) followed by 4% paraformaldehyde fixation solution [16,38]. The brains from the CE and RT groups were removed and immersed in a 4% paraformaldehyde solution for 48 h to achieve fixation. Then, the brain samples were immersed in a 30% sucrose solution for 24 h. The cerebral samples were serially sectioned into 30 μm coronal slices using a freezing microtome (CM1850, Leica, Wetzlar, Germany). A total of 10 slices was obtained from each brain and subsequently preserved at a temperature of −80 °C until analysis [16,38]. For Western blotting and q-PCR analysis, the hippocampus was isolated, washed in ice-cold normal saline, and stored at −80 °C until further use [16]. For the ubiquitinome analysis, the RT and CE groups each contained three replicates, with each replicate consisting of four mice. Brain samples were frozen in liquid nitrogen until used for the ubiquitinome analysis. For PK enzyme activity and mitochondrial-complex activity measurements, fresh hippocampal samples were used for detection. For Golgi staining, brain tissues of three RT mice and three CE mice were isolated and immersed in the tissue impregnation solution provided by the Golgi staining kit. Table 1 shows the number of mouse biological replicates used in the different methods in this study.

### 2.3. Immunohistochemistry

The brain sections (30 μm) were initially washed with PBS for 5 min. Subsequently, they underwent a 15 min 0.3% H_2_O_2_ treatment, followed by another round of PBS rinsing for 5 min (repeated three times). After that, the sections were blocked at room temperature for 10 min using goat serum albumin (Solarbio, Beijing, China) [37]. Then, the sections were incubated with a MAP-2 (#17490-1-AP, 1:100, Proteintech, Wuhan, China) primary antibody overnight at 4 °C [37]. Subsequently, the sections underwent a rinsing process with PBS for a duration of 5 min (repeated three times), followed by an incubation with the secondary antibody (HRP-labeled goat anti-rabbit IgG(H+L), A0208, 1:50, Beyotime, Shanghai, China) for 1 h at room temperature [37]. DAB (DA1010, Solarbio, Beijing, China) was used as a chromogenic agent. In the next step, the sections underwent dehydration by employing an alcohol gradient, followed by clearing in xylene.

Optical-density analysis of the immunohistochemical results was performed using IHC Toolbox, a plugin of the Image J software (version 1.53c). The “H-DAB” color-detection model was used for analysis.

### 2.4. Western Blotting

Hippocampal tissues were homogenized in RIPA buffer (P0013B, Beytime, Shanghai, China) containing PMSF (ST505, Beytime, Shanghai, China). The protein concentration was quantified utilizing the Enhanced BCA Protein Assay Kit (P0009, Beytime, Shanghai, China), following the instructions provided by the manufacturer. The Western blotting procedure was performed by following the previously reported methodology [14,16]. The details regarding the primary antibodies used in this investigation are provided in Table 2.

### 2.5. q-PCR Analysis

Total RNA was isolated from the hippocampus using the TRIzol reagent (Invitrogen, Carlsbad, CA, USA). For reverse transcription, cDNA was synthesized with the Transcriptor First Strand cDNA Synthesis Kit (Roche, Mannheim, Germany) according to the manufacturer’s instructions. Q-PCR was performed with the cDNA using TB Green Premix Ex Taq™ (TaKaRa, Beijing, China) according to the manufacturer’s instructions.

Primer sequences were as follows: for *Fh1*, TGTTACCGTTGGAGGCAGCAATG (FORWARD) and GTCTGTGAAGGACACTGAAGCATCTC (REVERSE); for *Sucla2*, TAAATGGTGCTGGCTTGGCTATGG (FORWARD) and ACGCTTCTGTTACTTGCTGGACTG (REVERSE); for *Suclg2*, CAGCGAACTTCTTGGACCTTGGAG (FORWARD) and TCCGTTGGCAATGATGGCACAG (REVERSE); for *Suclg1*, TTGTGTATTGGCATTGGAGGTGACC (FORWARD) and CTGTGGCTGGATCATTCAGGAAGAC (REVERSE); for *Ndufs1*, TGGGAACAACAGGAAGAGGAAATGAC (FORWARD) and GGCAGTAAAGGCATAAGGCTTAGAGG (REVERSE); for *Ndufv1*, AGAAGAGACGGCACTTATTGAA (FORWARD) and ATTTGGTACCTGAATTGCGTTC (REVERSE); and for *Ndufv2*, GGCTACCTATCTCCGCTATGAACAAG (FORWARD) and ATATGGTACTTCCCAACTGGCTTTCG (REVERSE). Relative mRNA levels were normalized to β-actin (Sangon Biotech, Shanghai, China), and the relative expression levels were calculated using the 2^−ΔΔCt^ method [37].

### 2.6. Golgi Staining

Golgi staining was performed using a HITO Golgi-Cox Optim Stain TM Prekit (Hitobiotec Corp, Wilmington, DE, USA) according to the manufacturer’s instructions. Briefly, mice were anesthetized and sacrificed. The brain tissues were removed and rinsed with double-distilled water. Then, the brain samples were transferred into tissue impregnation solution (equal volumes of solutions 1 and 2). The impregnation solution was replaced after 24 h, and samples were stored at room temperature in the dark for 14 days. Then the samples were transferred into solution 3. Solution 3 was replaced after 12 h, and the brain tissues were stored at 4 °C in the dark for 72 h. The brain tissues were frozen and then cut into 100 μm sections using a freezing microtome (CM1850, Leica, Wetzlar, Germany). A few drops of solution 3 were added to gelatin-coated slides, and sections were transferred to the gelatin-coated slides. After removing excess solution 3, the slides were air-dried overnight in the dark. Then, the slides were rinsed in distilled water 2 times for 3 min each. Tissue sections were placed in a mixed solution consisting of solutions 4 and 5 and double-distilled water, and these were then stained for 10 min. After being rinsed in distilled water two times (5 min each time), the sections were dehydrated with gradient alcohol and cleared in xylene. Golgi-stained neurons were observed using a laser scanning confocal microscope (TCS SP2, Leica), and Z-Stack technology was used to scan each segment (Z-step size 0.3 μm). Dendritic spines were analyzed with Image J software (version 1.53c). In order to quantify the dendritic-spine density, 15 dendritic segments (20 μm in length) from each mouse were counted (in total, 45 dendrites per group).

### 2.7. Ubiquitinome Analysis

Each sample was ground in liquid nitrogen, and 800 μL SDT lysate was added. Then, the samples were sonicated on ice for 2 min. The samples were centrifuged at 4 °C, and supernatants were collected. Then, the protein concentrations were determined using the BCA assay [39]. 10 μg of protein from each group of samples was taken for SDS-PAGE analysis. For each sample, 10 mg of total protein was treated with 10 mM dithiothreitol (DTT) for 1 h at room temperature to reduce disulfide bonds [39]. Then, the samples were treated with 50 mM iodoacetamide (IAA) and incubated for 30 min at room temperature in the dark. After adding urea (UA) buffer, the protein was precipitated with cold acetone overnight at −20 °C. Samples were centrifuged at 12,000× *g* for 10 min at 4 °C, and the precipitate was collected and washed once with cold acetone. Then, the samples were centrifuged again and air-dried. After adding 100 µL 8 M UA buffer to reconstitute the precipitated protein, the UA was diluted to below 1 M with 50 mM NH_4_HCO_3_ [39]. The samples were digested with trypsin (the ratio of trypsin: protein was 1:25 (W:W)) at 37 °C for 16–18 h. Then, the digestion was stopped by adding 100% TFA. The samples were desalted using Sep-PAK (according to the instructions of the PTMScan^®^ Ubiquitin Remnant Motif (K--GG) Kit (Cell Signaling Technology, #5562)), followed by freeze-drying under vacuum [39].

Dried samples were dissolved in 1 × immunoaffinity purification solution (IAP); these were then mixed with the PTMScan^®^ Ubiquitin Remnant Motif (K-ε-GG) Antibody Bead Conjugate and incubated for 2 h [39]. Then, the beads were washed with 1 × IPA and ultrapure water, and ubiquitinated peptides were eluted with 0.15% TFA. The eluted ubiquitinated peptides were collected and concentrated under vacuum, then purified using C18 tips (Thermo Scientific, Waltham, MA, USA) [39]. The purified peptide was lyophilized and dissolved in 10 µL of 0.1% FA for mass spectrometry analysis.

Chromatographic separation of each sample was performed using the Easy-nLC 1200 chromatography system (Thermo Scientific, Waltham, MA, USA) [39]. The binary buffer system was as follows: (A) 0.1% formic acid; and (B) 0.1% formic acid, acetonitrile, and water (such that acetonitrile is 95%). The samples were injected onto the Trap Column (100 µm × 20 mm, 5 µm, C18, Dr. Maisch GmbH, Ammerbuch, Germany) and were separated on the chromatographic analysis column (75 µm × 150 mm, 3 µm, C18, Dr. Maisch GmbH) at a flow rate of 300 nL/min. The liquid-phase gradient was as follows: 0–3 min, B linear gradient from 2% to 7%; 3–48 min, B linear gradient from 7% to 35%; 48–53 min, B linear gradient from 35% to 90%; 53–60 min, B linear gradient maintained at 90%. After separation of the peptides, a Q-Exactive Plus mass spectrometer (Thermo Scientific, Waltham, MA, USA) was used for data-dependent acquisition (DDA) mass spectrometry analysis. The MS scan properties were as follows: resolution: 70,000 at 200 *m*/*z*; AGC target: 1E6; maximum IT: 50 ms. The MS/MS scan properties were as follows: resolution: 17,500 at 200 *m*/*z*; AGC target: E5; maximum IT: 50 ms; MS2 activation type: HCD; isolation window: 2.0 Th; normalized collision energy: 27. 

The proteome data were analyzed using MaxQuant (version 1.6.0.16). The protein database used was UniProt Mus musculus (Mouse)-87867-20200113.fasta, containing a total of 87,867 protein sequences.

### 2.8. Metabolomic Analysis

The samples were homogenized at low temperature, then 200 μL of water and 800 μL of 1:1 methanol/acetonitrile were added to the samples, and the samples were sonicated for 60 min. To precipitate proteins, the samples were incubated for 1 h at −20 °C, followed by centrifugation for 20 min at 16,000× *g* and 4 °C. The corresponding amount of the internal standard L-Glutamate-d5 was added according to the weight of each sample, and then the samples were dried under vacuum. For mass spectrometry detection, the samples were resuspended in 100 μL of acetonitrile/water (1:1, *v*/*v*) and centrifuged at 16,000× *g* for 20 min at 4 °C, and the supernatant was collected for analysis.

The samples were separated by Shimadzu Nexera X2 LC-30AD high-performance liquid chromatography (Shimadzu, Kyoto, Japan). The mobile-phase components are shown in Table 3.

The sample was redissolved in 100 μL of 50% methanol aqueous solution. The column temperature was set to 40 °C, and the injection volume was 5 μL, with the flow rate set to 300 μL/min. The relevant liquid-phase gradient is shown in Table 4.

The mass spectrometry analysis was performed using a QTRAP5500 mass spectrometer (AB SCIEX, Beijing, China) in positive/negative-ion mode [40]. The ESI source parameters were set according to Table 5.

### 2.9. Mitochondrial-Complex Activity Measurements

The activities of mitochondrial complex I and complex IV of the hippocampus tissue were detected using the Mitochondrial Respiratory Chain Complex I and IV Activity Assay Kit (Solarbio, Beijing, China) according to the manufacturer’s instructions. The absorbance of the reaction mixture was determined by spectrophotometry.

### 2.10. PK Enzyme-Activity Measurements

The enzymatic activity of PK was determined by the PK Activity Assay Kit (Solarbio, Beijing, China) according to the manufacturer’s instructions.

### 2.11. Co-Immunoprecipitation Analysis

Immunoprecipitation was conducted using Protein A/G Dynabeads (88802, Thermo Fisher Scientific, Waltham, MA, USA) according to the manufacturer’s instructions. Briefly, hippocampal tissue was extracted using NP-40 Lysis Buffer (P0013F, Beytime, Shanghai, China). The hippocampal tissue lysates were incubated with a PSD-95 antibody at 4 °C overnight with mixing to form immune complexes. After pre-washing with TBST, magnetic beads were added to the immune complex and incubated at room temperature for 1 h with mixing. The beads were collected with a magnetic stand, washed with TBST three times, and then washed again with purified water. To elute the antigen–antibody complex, 100 μL of 0.1M pH 2.0 glycine solution was added and incubated for 10 min with mixing. The beads were magnetically separated, and the supernatant containing the target antigen was saved. To neutralize the low pH, 10 μL of 1M pH 8.5 Tris solution was added to each 100 μL of eluate.

### 2.12. Statistical Analysis

Statistical analyses utilized the Prism 8.0 software (Graphpad Software, San Diego, CA, USA). The data are presented as the mean ± standard deviation (SD). Differences were analyzed using unpaired Student’s *t*-tests (comparison between two groups), with *p* < 0.05 being considered significant.

## 3. Results

### 3.1. The Negative Effects of Cold Exposure on Hippocampal Neurodevelopment

**Figure 1:**
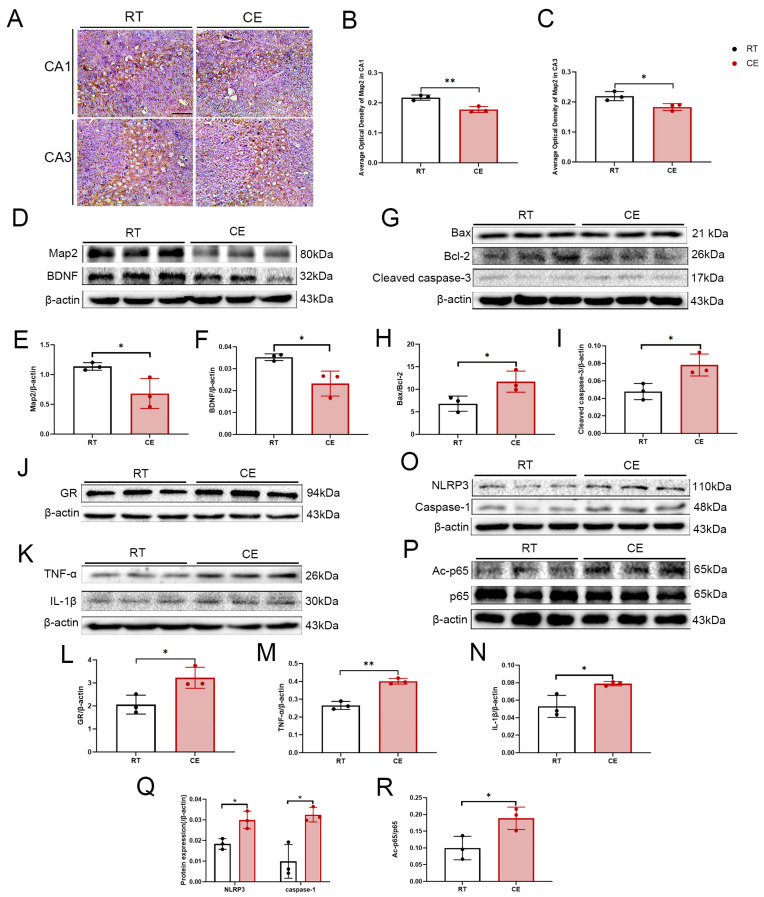
Negative effects of cold exposure on hippocampal neurodevelopment in adolescent mice. (**A**) Representative images of MAP-2 immunohistochemistry of the hippocampi of control and cold-exposure mice. Bar = 50 μm. (**B**,**C**) Graphs depicting the average optical-density analysis of MAP-2 immunostaining of hippocampal sub-regions CA1 and CA3. (**D**) Western blot demonstrating the levels of MAP-2 and BDNF in the hippocampi of mice from the CE and RT groups. Graphs indicate the results of densitometric analyses of the expression ratio of (**E**) MAP-2/β-actin and (**F**) BDNF/β-actin. (**G**) Western blot demonstrating the levels of Bax, Bcl-2, and cleaved-caspase-3 in the hippocampi of mice from the CE and RT groups. The graph indicates the results of densitometric analyses of the expression ratio of (**H**) Bax/Bcl-2 and (**I**) cleaved-caspase-3/β-actin. (**J**) Expression of GR was assessed by Western blotting. (**K**) Expression of the inflammatory cytokines TNF-α and IL-1β in the hippocampus after cold exposure were assessed by Western blotting. (**O**) Expression of NLRP3, caspase-1 in the hippocampus after cold exposure were assessed by Western blotting. (**P**) Expression of Ac-p65 and p65 in the hippocampus after cold exposure was assessed by Western blotting. The graphs indicate the results of densitometric analyses of the expression ratios of (**L**) GR/β-actin, (**M**) TNF-α/β-actin, (**N**) IL-1β/β-actin, (**Q**) NLRP3/β-actin and Caspase-1/β-actin, and (**R**) Ac-p65/p65. Results are expressed as means ± SD; n = 3. * *p* < 0.05; ** *p* < 0.01.

The distribution of microtubule-associated protein 2 (MAP-2) in the hippocampus was assessed by immunohistochemistry (Figure 1A). Cold exposure reduced the density of MAP-2 in the CA1 and CA3 regions of the hippocampus compared with the control (Figure 1B,C). Western blotting confirmed the reduced MAP-2 expression (Figure 1D,E). These results suggest that cold exposure inhibits the expression of the key protein for dendrite development, MAP-2. Moreover, expression of brain-derived neurotrophic factor (BDNF), an important neurotrophic factor, was significantly reduced following cold exposure (Figure 1D,F). Cleaved-caspase-3 protein levels and the Bax: Bcl-2 ratio were increased in the CE group (Figure 1G–I), indicating that cold exposure promotes apoptosis in the hippocampus.

Next, we detected the expression of the glucocorticoid receptor (GR) by Western blotting. The expression of GR was significantly upregulated following cold exposure, suggesting that glucocorticoid-receptor signaling is involved in the hippocampal response to cold exposure (Figure 1J,L). Moreover, the inflammatory cytokines tumor necrosis factor-α (TNF-α) and interleukin-1β (IL-1β) were significantly upregulated in the CE group (Figure 1K,M,N). Furthermore, the inflammatory regulator NOD-like-receptor thermal-protein-domain-associated protein 3 (NLRP3) and caspase-1 protein levels were significantly upregulated (Figure 1O,Q). The level of p65 Lys310 acetylation in the CE group was enhanced compared with the RT group (Figure 1P,R).

### 3.2. Ubiquitinome Profile of Proteins in the Hippocampus of Adolescent Mice following Cold Exposure

**Figure 2:**
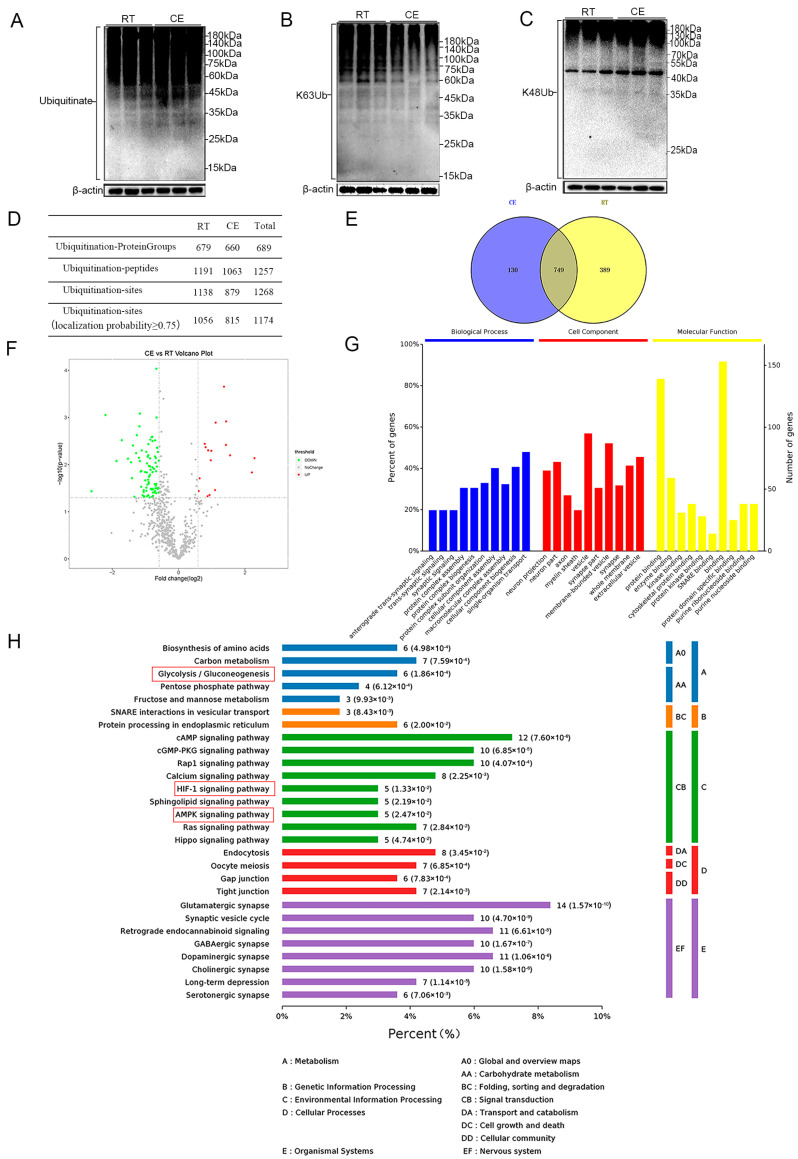
Ubiquitinome profiling of the hippocampi of RT and CE mice. (**A**) Western blot analysis of Ub species in hippocampal tissue. (**B**) Western blot analysis of K63-polyUb species in hippocampal tissue lysates. (**C**) Western blot analysis of K48-polyUb species in hippocampal tissue lysates. (**D**) The table shows the statistical analysis results of ubiquitination proteomics. (**E**) The Venn diagram shows the comparison of ubiquitination sites identified in CE vs. RT. (**F**) The volcano plot shows the log2-transformed fold change and log10-transformed *p*-value of the identified ubiquitination sites, highlighting significantly regulated lysine (K) sites. Significantly upregulated ubiquitination sites are displayed as red dots, and downregulated ubiquitination sites are displayed as green dots. (**G**) GO annotation of the ubiquitinome. (**H**) KEGG pathway analysis of the ubiquitinome.

To assess changes in ubiquitination within the hippocampus following cold exposure, we performed Western blot analyses. An increase was observed in global Ub protein levels following cold exposure (Figure 2A). The ubiquitin-chain type with the highest content in rat brain is the poly-K48 chain, and the second most abundant is the poly-K63 chain [41]. Functional studies of these two classical ubiquitin chains are more thorough than for other non-classical ubiquitin chains [24]. To further detect changes in K48-polyUb and K63-polyUb, linkage-specific antibodies against the K63-polyUb chains and K48-polyUb chains were used. The results showed that K63-polyUb smear bands decreased following cold exposure (Figure 2B). K48-polyUb smear bands increased in the CE group compared with the RT group (Figure 2C).

We conducted an analysis of the ubiquitination landscape of the hippocampus in adolescent mice after chronic cold exposure. We identified a total of 1268 ubiquitination sites on 1257 ubiquitinated peptides (Figure 2D) (Supplemental Table S1). As shown in the Venn diagram, there were 130 sites in the cold-exposure group, 389 sites in the control group, and 749 sites in both groups (Figure 2E). Among these, 1174 ubiquitination sites meet the condition of localization probability ≥ 0.75 and were considered as reliable sites. Sites that meet the conditions of *p* < 0.05 and fold change > 1.5 were judged as significant differential ubiquitination sites (Figure 2F, Supplemental Table S2). In order not to miss putatively interesting candidates, we also considered the possibility of extreme differences between the RT and CE groups [42]. Therefore, we also investigated diGly sites that were exclusively identified in two or three out of three replicates in one group while not being identified in any of the three replicates in the other group [42]; this was considered a case of extreme differences (Supplemental Table S3).

To understand the biologically functional role of ubiquitination, we annotated proteins by Gene Ontology (GO) enrichment analysis. The significantly enriched GO terms in the biological process, cellular component, and molecular function categories mainly included trans-synaptic signaling, synaptic signaling, neuron projection, synapse part, axon, synapse, and soluble N-ethylmaleimide-sensitive factor attachment-protein receptor (SNARE) binding (Figure 2G). To decipher the cellular pathways in which the proteins with differentially ubiquitinated sites are involved, we performed a KEGG enrichment analysis. The following pathways were identified: synaptic vesicle cycle, glutamatergic synapse, and GABAergic synapse, among others. In particular, proteins with differential ubiquitinated sites induced by cold exposure were significantly enriched in the glycolysis/gluconeogenesis pathway, the pentose phosphate pathway, and fructose and mannose metabolism (Figure 2H). In addition, classical cellular metabolic-regulation pathways were identified, such as the HIF-1 signaling pathway and the AMPK signaling pathway (Figure 2H). These data indicate that proteins with differentially ubiquitylated residues induced by cold exposure may participate in synaptic activities and glucose metabolism processes in the hippocampus. 

### 3.3. Chronic Cold Exposure Affects PSD-95 Ubiquitination within the Hippocampus

**Figure 3:**
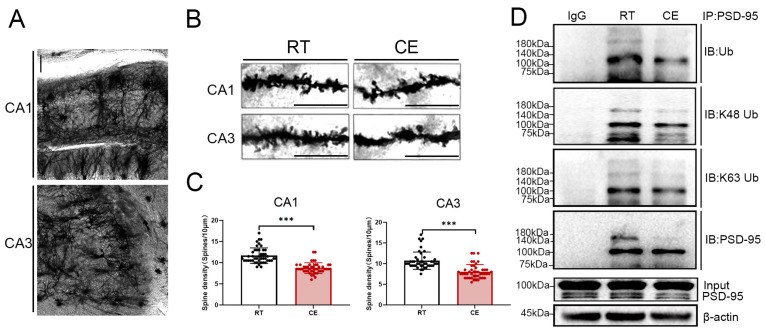
Cold exposure downregulates the density of dendritic spines and PSD-95 ubiquitination levels within the hippocampus. (**A**) A representative low magnification image of Golgi–Cox staining in the hippocampus. Bar = 100 μm. (**B**) Representative images of dendritic spines in the hippocampal CA1 and CA3 subregions from RT and CE mice. Bar = 10 μm. (**C**) Quantification of dendritic spines per 10 μm of the hippocampal CA1 and CA3 subregions from RT and CE mice. n = 45 dendritic segments from 3 animals per group. (**D**) Immunoprecipitation of PSD-95 from the hippocampi from RT and CE mice. Values are presented as the mean ± SD (n = 3). Statistically significant differences are indicated: *** *p* < 0.0001.

The proteomic analysis identified a variety of differentially ubiquitinated synapse-related proteins. Golgi staining was used to assess the effects of cold exposure on dendritic-spine density within the hippocampus. As shown in Figure 3, cold exposure significantly reduced dendritic-spine density in the hippocampal sub-regions CA1 and CA3 (Figure 3A–C).

The ubiquitinome analysis demonstrated that the ubiquitination levels of PSD-95 at residues K202, K558, and K591 were significantly reduced after chronic cold exposure. Immunoprecipitation was used to assess the ubiquitination level of PSD-95, which confirmed that the ubiquitination level of PSD-95 was significantly reduced following cold exposure (Figure 3D). It is known that K48-linked polyubiquitin-chain attachment results in substrate proteasome-mediated degradation [28]. A recent study demonstrated K63-linked ubiquitination of PSD-95 at residue K558 to be the basal mechanism by which PSD-95 promotes synaptic maturation [32]. Therefore, it was necessary to distinguish the polyubiquitin-chain types attached to PSD-95. Specific antibodies targeting K48-linked and K63-linked polyubiquitin chains were used, and chronic cold exposure was shown to decrease both the K63-linked and K48-linked ubiquitination of PSD-95 (Figure 3D). Collectively, our results demonstrated that cold exposure reduced dendritic-spine density within the hippocampus and decreased ubiquitination of key functional residue K558 of PSD-95. 

### 3.4. Chronic Cold Exposure Induces Changes in the Glycolysis Pathway within the Hippocampus

**Figure 4:**
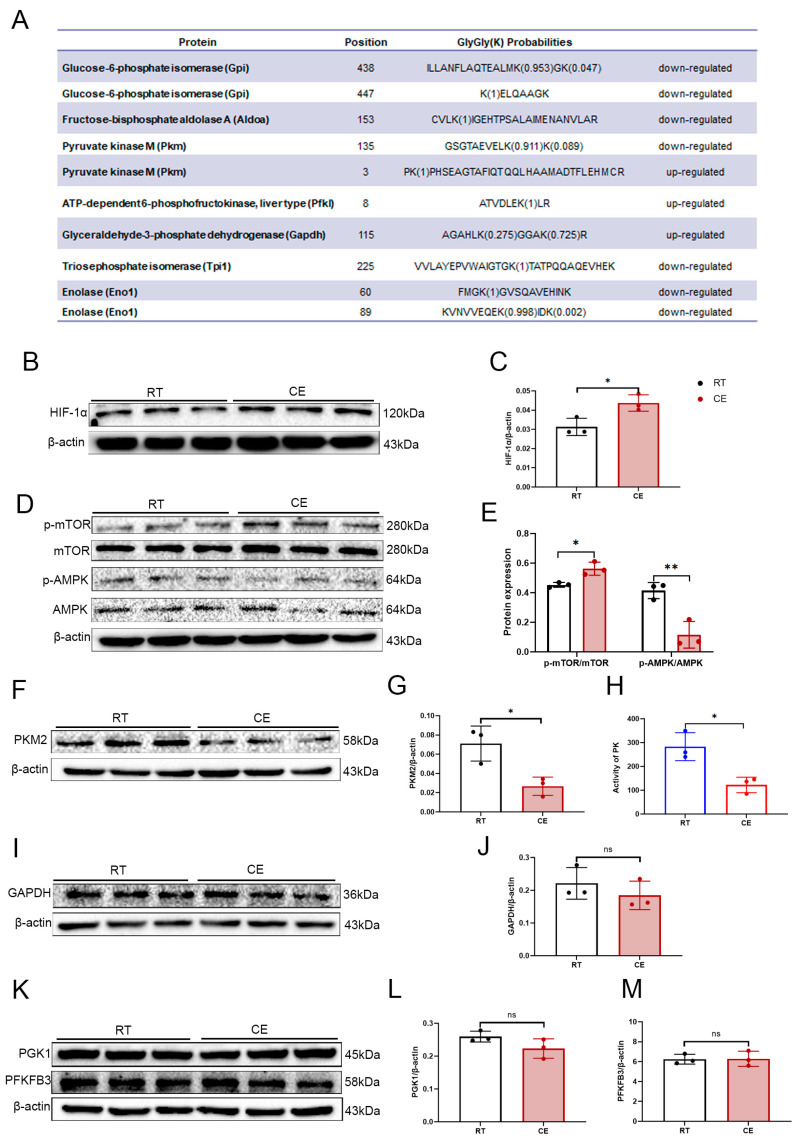
The effect of cold exposure on a variety of glycolytic enzymes. (**A**) Glucose-metabolizing enzymes with differential ubiquitination sites. (**B**) The expression of HIF-1α was analyzed by Western blotting. (**C**) The graph indicates the expression ratios of HIF-1α/β-actin obtained from the densitometric analyses. (**D**) The expression of p-mTOR, mTOR, p-AMPK, and AMPK in the hippocampal homogenates. (**E**) The graphs indicate the expression ratios of p-mTOR/mTOR and p-AMPK/AMPK based on the densitometric analyses. (**F**) The expression of PKM2 was analyzed by Western blotting. (**G**) The graph indicates the expression ratios of PKM2/β-actin based on the densitometric analyses. (**H**) Enzyme activity of PK within the hippocampi of RT and CE mice. (**I**) The expression of GAPDH was analyzed by Western blotting. (**J**) The graph indicates the expression ratios of GAPDH/β-actin based on the densitometric analyses. (**K**) The expression of PGK1 and PFKFB3 in the hippocampal homogenates. The graphs indicate the expression ratios of (**L**) PGK1/β-actin and (**M**) PFKFB3/β-actin based on the densitometric analyses. Values are presented as the mean ± SD (n = 3). Statistically significant differences are indicated: * *p* < 0.05, ** *p* < 0.01; ns: not significant.

Herein, a series of glucose metabolic enzymes were found to have differentially ubiquitinated sites, including glucose-6-phosphate isomerase (GPI) (K438, K447), fructose-bisphosphate aldolase A (ALDOA) (K153); pyruvate kinase M (PKM) (K3, K135); ATP-dependent 6-phosphofructokinase, liver type (PFKL) (K8); glyceraldehyde-3-phosphate dehydrogenase (GAPDH) (K115); triosephosphate isomerase (TPI) (K225); and enolase 1 (ENO 1) (K60, K89) (Figure 4A). This series encompasses essentially all the steps of glycolysis. The KEGG pathway enrichment analysis of differentially ubiquitinated proteins also found significant enrichment in the HIF-1 signaling pathway and the AMPK signaling pathway. HIF-1α is an important transcription factor that promotes cellular glycolysis by regulating the transcription of genes encoding glycolytic enzymes, including PKM2, lactate dehydrogenase A (LDHA), GAPDH, hexokinase (HK), PFKL, ENO1, and ALD [43,44,45]. Further, mTOR acts as an upstream activator of HIF-1α in the regulation of cellular glucose metabolism [46]. AMPK, the main sensor of cellular energy status, has been reported to inhibit the phosphorylation of mTOR [47]. Accordingly, the expression of the AMPK/mTOR/HIF-1a pathway was examined to deeply explore the impact of cold exposure on HIF-1α and its regulator. Western blot analysis demonstrated a dramatic increase in HIF-1α levels following cold exposure (Figure 4B,C). The phosphorylation level of mTOR was also significantly increased, while the phosphorylation level of AMPK was decreased (Figure 4D,E), suggesting that the mTOR/HIF-1α pathway in the hippocampus is activated by cold exposure.

The ubiquitinome results in this study identified that PKM underwent significant differential ubiquitination following cold exposure. PKM is the third rate-limiting enzyme, as well as the second ATP-generating enzyme, in the glycolytic pathway [48]. RNA sequencing data from Zhang et al. [49] combined with Western blotting and immunohistochemical evidence from Wei et al. [50] confirmed that neurons mainly express PKM1, while astrocytes mostly express PKM2. It is also known that under normal conditions, the glycolytic flux of astrocytes in the brain is higher than that of neurons. The lactic acid produced by astrocyte glycolysis can provide energy for neuron activity through “the lactic acid shuttle” between astrocytes and neurons, which is an important way for neurons to be energized [51]. Therefore, we evaluated the effects of cold exposure on PKM2. Western blots demonstrated that cold exposure markedly reduced the expression of PKM2 (Figure 4F,G). The enzymatic activity of PK was also reduced (Figure 4H).

As shown by the ubiquitinome results, GAPDH has significant differential ubiquitination sites. In the glycolysis pathway, there are two steps that can directly produce ATP, and the GAPDH/phosphoglycerate kinase (PGK) complex is the first ATP-generating step [48,52]. Although GAPDH can be used as a loading control, the important function of GAPDH should not be ignored. In neurons, the GAPDH/PGK located in vesicles can provide enough ATP for rapid axonal transport (FAT) of vesicles by specifically localized glycolytic machinery, independent of mitochondria [53]. Furthermore, GAPDH/PGK complexes located at the PSD and synaptic vesicles also can supply ATP for synaptic transmission activity by the localized glycolytic machinery [54,55]. Given the important role of the GAPDH/PGK complex in neuronal function, we next tested the expression of GAPDH and PGK in the hippocampus. The results showed that cold exposure did not cause changes in GAPDH (Figure 4I,J) and PGK1 expression (Figure 4K,L).

It is reported that AMPK can regulate glycolysis through 6-phosphofructo-2-kinase/fructose-2,6-bisphosphatase 3 (PFKFB3) [56,57]. PFKFB3 plays a key role in regulating the activity of PFK, the second rate-limiting enzyme in the glycolysis pathway, by controlling the synthesis and degradation of fructose-2,6-diphosphate, the strongest allosteric activator of PFK [58,59]. In addition, PFKFB3 specifically exhibits high activity in astrocytes. In neurons, PFKFB3 is continuously degraded by the ubiquitin–proteasome mediated by the E3 ubiquitin ligase anaphase promoting complex/ cyclosome (APC/C)-Cdh1 [59,60]. Thus, we tested PFKFB3 expression. The results showed that PFKFB3 expression was not significantly altered following cold exposure (Figure 4K,M).

### 3.5. Chronic Cold Exposure Induces Changes in the Glucose Aerobic Oxidation Pathway within the Hippocampus

**Figure 5:**
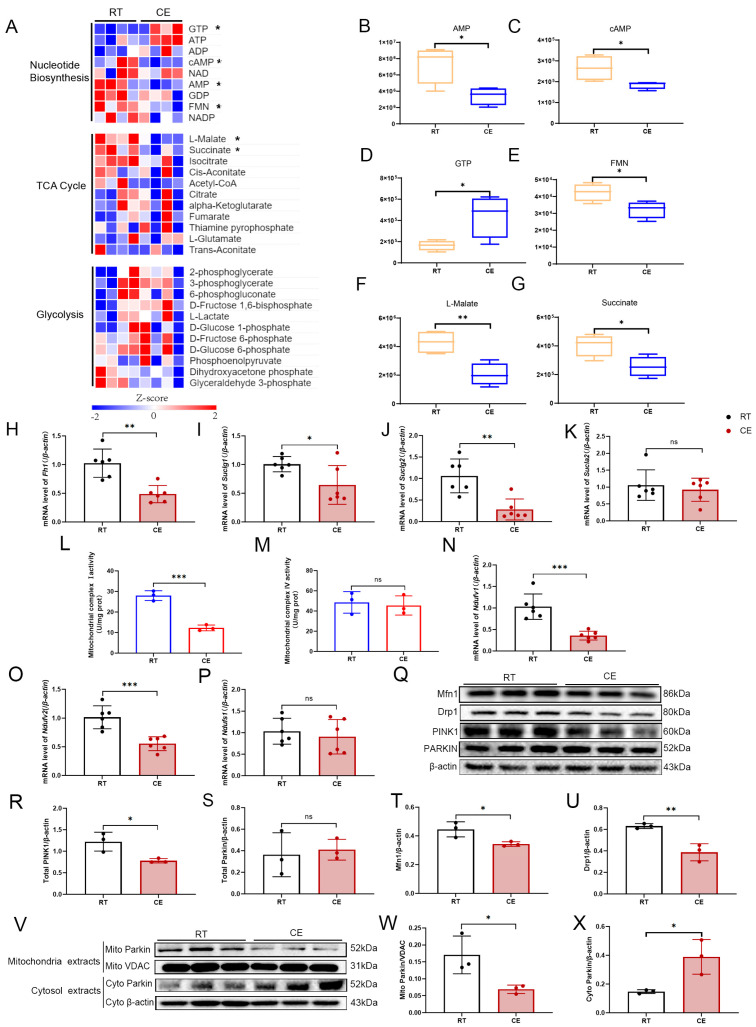
Cold exposure affects the tricarboxylic acid cycle and mitochondrial function within the hippocampus. (**A**) Heat map showing the energy metabolomics within the hippocampi of RT and CE mice. Box diagrams depicting the content of (**B**) AMP, (**C**) cAMP, (**D**) GTP, (**E**) FMN, (**F**) L-malate, and (**G**) succinate in energy metabolomics. Values are presented as the mean ± SD (n = 4). Statistically significant differences are indicated: * *p* < 0.05, ** *p* < 0.01. The mRNA levels of *Fh1* (**H**), *Suclg1* (**I**), *Suclg2* (**J**), *Sucla2* (**K**) in the hippocampi of RT and CE mice (n = 6). (**L**) Activity of mitochondrial complex I within the hippocampi of RT and CE mice (n = 3). (**M**) Activity of mitochondrial complex IV within the hippocampi of RT and CE mice (n = 3). (**N**) The mRNA levels of *Ndufv1* in the hippocampi of RT and CE mice (n = 6). (**O**) The mRNA levels of *Ndufv2* in the hippocampi of RT and CE mice (n = 6). (**P**) The mRNA levels of *Ndufs1* in the hippocampi of RT and CE mice (n = 6). (**Q**) Western blot showing the levels of Mfn1, Drp1, PINK1, PARKIN in the hippocampi of control and cold-exposure mice (n = 3). The graphs indicate the expression ratio of (**R**) PINK1/β-actin, (**S**) PARKIN/β-actin (n = 3), (**T**) Mfn/β-actin, and (**U**) Drp1/β-actin based on densitometric analyses. (**V**) Western blot showing the levels of PARKIN in the mitochondrial component and in the cytoplasmic component of the hippocampus for control and cold exposure mice (n = 3). The graphs indicate the expression ratios of (**W**) Mito Parkin/VDAC, (**X**) Cyto Parkin/β-actin (n = 3) based on densitometric analyses. Values are presented as the mean ± SD. Statistically significant differences are indicated: * *p* < 0.05, ** *p* < 0.01, *** *p* < 0.0001; ns: not significant.

A metabolomics study was conducted to explore the exact alterations in glucose metabolism processes induced by cold exposure (Figure 5A). ATP levels were not significantly changed, while AMP levels were significantly downregulated, suggesting that cold exposure disrupts the rate balance between ATP production and hydrolysis (Figure 5B). The content of flavin mononucleotide (FMN), an important cofactor of mitochondrial respiratory complex I, was decreased (Figure 5E). In addition, chronic cold exposure selectivity reduced the level of L-malate and succinate, important metabolites of the TCA cycle (Figure 5F,G). Next, we assessed the gene expression of fumarate hydratase and succinyl coenzyme A synthase. We observed decreased mRNA levels of fumarate hydratase 1 (*Fh1*); succinate-CoA ligase, GDP-forming, subunit alpha (*Suclg1*); and succinate-CoA ligase, GDP-forming, subunit beta (*Suclg2*) (Figure 5H–J). These data indicate that cold exposure selectively impairs the generation of L-malate and succinate during the TCA cycle in the hippocampus.

Mitochondria are central to the control of cellular energy metabolism. The activities of mitochondrial respiratory complex I (CI), the initial enzyme in the electron-transfer chain, and mitochondrial respiratory complex IV (CIV), the terminal oxidase in the electron-transfer chain, were determined [61]. Cold exposure resulted in a significant decrease in the activity of mitochondrial respiratory complex I; however, the activity of mitochondrial respiratory complex IV was not altered (Figure 5L,M).

Mitochondrial respiratory complex I, also known as NADH: ubiquinone oxidoreductase, is a flavoenzyme with FMN as the primary electron receptor [62]. The NADH dehydrogenase module (N module) of mitochondrial respiratory chain complex I is responsible for binding to and oxidizing NADH. NDUFV1, NDUFV2, and NADH ubiquinone oxidoreductase core subunit S1 (NDUFS1) are the core subunits constituting the N module [62]. FMN binds closely to NDUFV1 in a non-covalent manner and directly receives electrons from NADH and transmits them to iron–sulfur (Fe-S) clusters [61,63]. Energy metabolomic results showed that cold exposure reduced the FMN content in the hippocampus. Thus, gene expression of *Ndufv1* and *Ndufv2* was detected. The mRNA levels of *Ndufv1* and *Ndufv2* (Figure 5N,O) were significantly reduced, while the mRNA level of *Ndufs1* (Figure 5P) was not changed, suggesting that impaired mitochondrial function caused by cold exposure is due to the reduced gene expression of *Ndufv1* and *Ndufv2*.

KEGG analysis of ubiquitination proteomics revealed that differentially ubiquitinated proteins in the AMPK signaling pathway were enriched. Moreover, AMPK regulates mitophagy and mitochondrial dynamics in response to changes in energy supply [64]. Thus, after observing the negative impact of cold exposure on mitochondrial function, we wondered if the mitochondrial quality-control pathway is also affected by cold exposure. It has been reported that under energetic stress, AMPK promotes PARKIN-mediated mitophagy by phosphorylating UNC-51-like kinases 1 (ULK1) [65]. Therefore, we determined the expression of PTEN-induced putative kinase 1 (PINK1) and PARKIN. The results showed that PINK1 was significantly downregulated following cold exposure, while the protein expression of PARKIN was not changed (Figure 5Q–S). Next, we isolated mitochondrial and cytoplasmic components of the hippocampus and assessed the expression of PARKIN. The results showed that chronic cold exposure downregulated the transposition of PARKIN to the mitochondria (Figure 5V–X), suggesting that the PINK1/PARKIN-mediated mitochondrial quality-control pathway was inhibited by chronic cold exposure. Taken together, these results demonstrate that chronic cold exposure inhibits the function of mitochondrial respiratory complex I and interferes with mitochondrial quality-control through PINK1/PARKIN pathway. Mitophagy is coupled with mitochondrial dynamics to complete the mitochondrial quality-control mechanism [66]. In addition, under mitochondrial stress, AMPK regulates dynamin-related protein 1 (Drp1)-mediated mitochondrial fission through the phosphorylation of mitochondrial fission factor (MFF) [67]. To evaluate the effect of cold exposure on mitochondria, the expression of mitochondrial fusion and division proteins was evaluated. The expression of mitofusin 1 (Mfn1) and Drp1 was significantly downregulated (Figure 5Q,T,U), suggesting that the mitochondrial fusion mediated by Mfn1 and the fission mediated by Drp1 are affected by cold exposure.

## 4. Discussion

**Figure 6:**
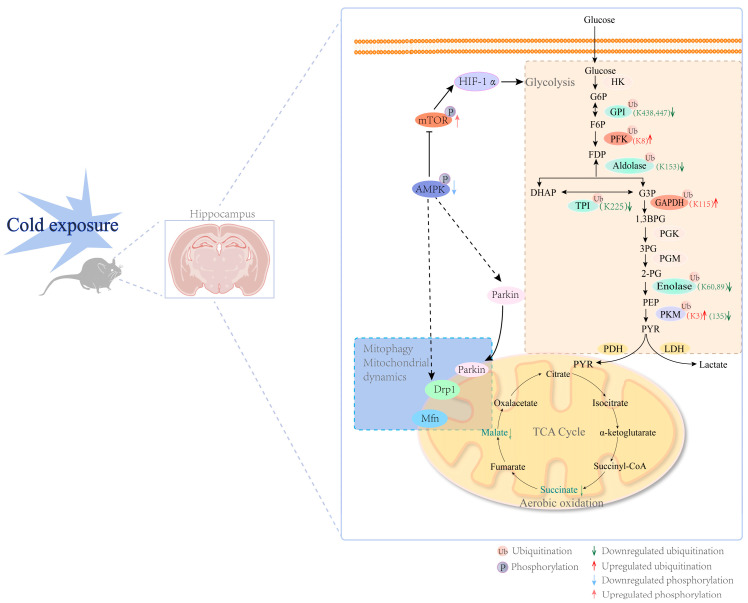
A schematic diagram of the effect of chronic cold exposure on hippocampal energy metabolism. A series of glucose metabolic enzymes were found to have differentially ubiquitinated sites, including GPI (K438, K447), ALDOA (K153), PKM (K3, K135), PFKL (K8), GAPDH (K115), TPI (K225), and ENO 1 (K60, K89). In the TCA cycle, cold exposure selectively impairs the generation of L-malate and succinate. Cold exposure also inhibited the activity of PK. The hippocampus image in Figure 6 was created with BioRender.com (accessed on 1 June 2022).

Herein, we have drawn a global picture of the cold-exposure regulation of hippocampal protein ubiquitination related to glucose metabolism (Figure 6). Cold exposure significantly decreased the density of dendritic spines and the level of PSD-95 ubiquitination. Moreover, we demonstrated chronic cold exposure to be a threat to hippocampal energy homeostasis. Chronic cold exposure causes energy homeostasis dysregulation within the hippocampi of adolescent mice. A variety of glucose metabolic enzymes are mobilized to cope with the energy challenge posed by long-term cold exposure.

As demonstrated by McEwen, chronic stress remodels dendrites of the CA3 pyramidal neurons and reduces neurogenesis within the DG region, which are two well-known forms of stress-induced structural remodeling of the hippocampus [7,68]. In this study, we observed that chronic cold exposure for 21 days significantly reduced MAP-2 expression in the hippocampal CA1 and CA3 regions. This is consistent with the negative effects of stress on dendritic development observed in other psychological stress models [69,70]. MAP-2 is essential to dendritic growth in that it serves as a cross-linker between microtubules and neurofilaments of the neuronal cytoskeleton [71,72]; in addition, it anchors PKA to dendrites, promoting neuronal signal transmission [73]. Since MAP-2 expression was suppressed by cold exposure, we speculate that cold exposure may interfere with dendritic growth processes associated with MAP-2. Cold exposure and other chronic psychological stressors share, at least in part, similar neuroplasticity mechanisms such as the loss of hippocampal dendritic spines, as well as reductions in MAP-2 and BDNF abundance. Additionally, we also observed that cold exposure induced the upregulation of inflammatory and apoptotic proteins, suggesting that chronic cold exposure induces neuroinflammation and apoptotic responses in the hippocampus. Collectively, chronic cold exposure has significant negative effects on hippocampal neurodevelopment in adolescent mice.

Significantly, we observed a decrease in hippocampal dendritic-spine density after cold exposure. Similar results have been reported for chronic unpredictable mild stress, chronic social-defeat stress, and restraint stress [74,75,76,77]. Our ubiquitinomic analysis revealed that the ubiquitination levels of PSD-95 at residues K202, K558, and K591 were significantly decreased after chronic cold exposure. Our immunoprecipitation results further validated the results observed with the ubiquitinomic analysis. In subsequent immunoblotting assays, we observed reduced PSD-5 ubiquitination when ubiquitination antibodies, K63-chain-specific ubiquitination antibodies, and K48-chain-specific ubiquitination antibodies were used. The synaptic-activity regulatory function of PSD-95 is highly correlated with its ubiquitination, and both proteasome-dependent and proteasome-independent functions of PSD-95 ubiquitination have been reported [78,79]. In 2016, Ma et al. found that the K558 residue of PSD-95 was specifically ubiquitinated with K63-linkage ubiquitin, which was crucial for PSD-95 to target dendritic spines, interact with the postsynaptic scaffold proteins, and support and stabilize surface AMPAR receptors [32]. A K558R mutant rendered PSD-95 incapable of promoting synaptic maturation in vivo and in vitro [32]. The study conducted by Ma et al. revealed that transfection of PSD-95 wild-type (WT), rather than the PSD-95 K558R mutant, led to a notable and significant restoration of dendritic-spine density in the hippocampal neurons of PSD-95 knockout (KO) mice [32]. In this study, ubiquitination proteomics identified the ubiquitination level of PSD-95 at the K558 residue to be dramatically downregulated after cold exposure, which may adversely affect the postsynaptic scaffold function of PSD-95.

Studies have reported that mitochondrial dysfunction is involved in stress-induced synaptic defects [21]. To explore the mechanisms underlying the negative effects of cold exposure on hippocampal neurodevelopment, we focused on changes in energy metabolism within the hippocampus. Importantly, bioinformatic analysis of the ubiquitinome revealed that cold-exposure-induced differentially ubiquitinated proteins were significantly enriched for the AMPK and HIF-1α pathways. We also found that cold exposure inhibited AMPK phosphorylation and promoted mTOR phosphorylation. Since AMPK has been proved to play a key role in regulating cellular energy homeostasis, including in mitochondrial activity and glycolysis [80], we hypothesized that the overall energy metabolism in the hippocampus is affected by cold exposure. We initially explored the effects of cold exposure on several steps in the aerobic oxidation of glucose and glycolysis within the hippocampus. First, we observed that chronic cold exposure exerted a negative effect on hippocampal mitochondria function. Cold exposure reduced the gene expression of *Ndufv1* and *Ndufv2*, key subunits of mitochondrial respiratory complex I. By affecting the Drp1 and PARKIN proteins, AMPK profoundly affects mitochondrial quality control [65,67]. Therefore, further investigation was carried out to examine the influence of cold exposure on PINK1 and PARKIN. Consequently, it was speculated that the normal clearance of damaged mitochondria was disturbed, in that PINK1/PARKIN pathway, the important mechanism that exert mitochondrial quality-control, was significantly inhibited. The normal expression and function of PINK1 is important for the nervous system to resist the negative effects of stress responses. It was reported that PINK1 deficiency inhibits adult hippocampal neurogenesis, and PINK1-knockout mice exhibit severe depressive behaviors under chronic restraint stress [81]. Moreover, cold exposure inhibits PARKIN translocation to the mitochondria. Inhibition of the PINK1/PARKIN pathway due to cold exposure may further hinder the recovery of the mitochondrial function in hippocampal neurons. Of interest, a recent study suggested that high-dose glucocorticoids induce mitochondrial damage that results in synapse defects in hippocampal neurons, and this is due to the impairment of NIX-mediated mitophagy but not the PINK1–PARKIN pathway [21]. Although it is well known that glucocorticoids mediate nerve damage during the stress response, the differences between exposure to cold and glucocorticoids suggest that chronic cold exposure may have unique features that differ from other psychological stressors.

With regard to the effect of cold exposure on glycolytic processes in the hippocampus, we observed that the enzyme activity of PK, the rate-limiting enzyme that catalyzes the last step of glycolysis, was significantly decreased. Metabolic intermediates participate in the regulation of the activity of PKM2. We found that chronic cold exposure did not alter the level of fructose 1, 6-bisphosphate (FBP), an important allosteric activator of PKM2, indicating that the cold-exposure-induced change in PKM activity may not be due to the regulation of FBP. It is important to note that protein post-translational modifications are the main regulatory means of PKM2 conformational transition as well as activity. Post-translational modifications, including acetylation and phosphorylation, have been reported to regulate PKM2 activity [82,83]. We observed that the ubiquitination of PKM at K3 was significantly up-regulated, while the ubiquitination of K135 was downregulated. But it has not been revealed whether there is a regulatory relationship between the ubiquitination of PKM and its enzyme activity. Furthermore, as shown by the metabolomics data, we observed that the balance between the synthesis and degradation of ATP and AMP was disrupted, although the ATP content was not changed significantly. These results suggested that the hippocampus is vulnerable and sensitive to subtle changes in the energy state caused by cold exposure, but the hippocampus also exhibits high potential to adapt to mild changes in energy demand.

Collectively, cold exposure poses a challenge to energy metabolism in the hippocampus. An important outcome of this study is the preliminary identification of the process by which chronic cold exposure impacts energy metabolism within the hippocampus. Specifically, cold exposure activated the mTOR/HIF-1α pathway in the hippocampus, affecting mitochondrial function, the TCA cycle, and glycolysis. Chronic cold exposure was shown to inhibit several important steps in glucose aerobic oxidation within the hippocampus. First, chronic cold exposure significantly affected the activity of mitochondrial respiratory complex I by inhibiting the gene expression of key subunits, *Ndufv1* and *Ndufv2*, and the FMN content. Finally, the steps catalyzed by FH and SUCL in the TCA cycle were inhibited by chronic cold stress. With regard to glycolysis, the ubiquitin modification levels of a variety of key enzymes in glucose metabolism were significantly changed. Chronic cold exposure suppressed PK enzymatic activity and the expression of PKM2. In general, cold exposure disrupted the metabolic homeostasis of the hippocampus. Although stress-induced nerve damage has been studied for decades, there remain a number of unanswered questions. Our work highlights the important role of ubiquitination in the response to stress. The profile of ubiquitinated proteins revealed by proteomics provides a large number of novel insights into the relationship between the stress response and neuronal function, for not only cold stress but also for other stress paradigms. In the future, the negative impact of stress may be alleviated by interventions at the level of protein ubiquitination.

## 5. Conclusions

Cold exposure exerted a negative impact on the development of dendritic spines in adolescent mice. The ubiquitination level of PSD-95 was significantly changed. Importantly, cold exposure induced abnormalities in energy homeostasis within the hippocampus, activating the mTOR/HIF-1α pathway and altering the ubiquitination levels of various glucose-metabolizing enzymes.

## Figures and Tables

**Table 1:** Biological replicates in various methods.

Method	Number of Mice (per Group)
Immunohistochemistry	n = 3
Western blotting	n = 3
q-PCR	n = 6
PK enzyme activity measurements	n = 3
Mitochondrial-complex activity measurements	n = 3
Metabolomic analysis	n = 4
Golgi staining	n = 3
Ubiquitinome analysis	n = 12

**Table 2:** Primary antibody information.

Primary Antibody	Catalog Number	Manufacturer	Concentration
NLRP3	15101	Cell Signaling Technology, Boston, MA, USA	1:1000
Acetyl-NF-κB p65 (Lys310)	12629	Cell Signaling Technology	1:1000
NF-κB P65	66535-1-Ig	Proteintech	1:1000
IL-1 Beta	26048-1-AP	Proteintech	1:1000
Bax	50599-2-Ig	Proteintech	1:1000
Bcl-2	60178-1-Ig	Proteintech	1:1000
BDNF	66292-1-Ig	Proteintech	1:1000
PARKIN	14060-1-AP	Proteintech	1:1000
PINK1	23274-1-AP	Proteintech	1:1000
VDAC1/2	10866-1-AP	Proteintech	1:1000
Mfn1	13798-1-AP	Proteintech	1:1000
Drp1	12957-1-AP	Proteintech	1:1000
Beta actin	60008-1-Ig	Proteintech	1:1000
Caspase-1	22915-1-AP	Proteintech	1:1000
PGK1	17811-1-AP	Proteintech	1:1000
PKM2	15822-1-AP	Proteintech	1:1000
Caspase-3	22915-1-AP	Proteintech	1:1000
PSD-95	20665-1-AP	Proteintech	1:1000
TNF-α	17590-1-AP	Proteintech	1:1000
AMPKα	5831	Cell Signaling Technology	1:1000
Phospho-AMPK (Thr172)	50081	Cell Signaling Technology	1:1000
Phospho-mTOR (Ser2448)	5536	Cell Signaling Technology	1:1000
mTOR	2983	Cell Signaling Technology	1:1000
Anti-Ubiquitin (linkage-specific K63) [EPR8590-448]	ab179434	Abcam, Shanghai, China	1:1000
Anti-Ubiquitin (linkage-specific K48) [EP8589]	ab140601	Abcam	1:1000
Anti-Ubiquitin	3933S	Cell Signaling Technology	1:1000
PFKFB3	13763-1-AP	Proteintech	1:1000
MAP-2	17490-1-AP	Proteintech	1:1000
HIF-1α	36169	Cell Signaling Technology	1:1000
GAPDH	AC002	ABclonal, Wuhan, China	1:1000

**Table 3:** Mobile-phase components.

Mobile-Phase A	Mobile-Phase B
5% acetonitrile aqueous solution	95% acetonitrile aqueous solution
10 mM ammonium acetate	10 mM ammonium acetate
pH 9	pH 9

**Table 4:** Relevant liquid-phase gradients.

Time	Liquid-Phase Gradient
0–2 min	95%B
2–9 min	95% to 70%B
9–10 min	70–30%B
10–11 min	30%B
11–11.5 min	30% to 95%B
11.5–15 min	95%B

**Table 5:** ESI source parameters.

Positive-Ion Mode	Negative-Ion Mode
Source Temperature: 550 °C	Source Temperature: 550 °C
Ion-Source Gas1 (GAS1): 40	Ion-Source Gas1 (GAS1): 40
Ion-Source Gas2 (GAS2): 50	Ion-Source Gas2 (GAS2): 50
Curtain Gas (CUR): 35	Curtain Gas (CUR): 35
Ion-Spray Voltage Floating (ISVF): 5500 V	Ion-Spray Voltage Floating (ISVF): −4500 V

## Data Availability

The data supporting this article will be shared by the corresponding author upon reasonable request.

## Supplementary Materials

The following supporting information can be downloaded at: https://www.mdpi.com/article/10.3390/cells13070570/s1. Table S1. GlyGly (K) sites; Table S2. Quantitative and differential analysis of the ubiquitinome data; Table S3. GlyGly (K) sites exclusively identified in one group.
